# Passive exercise to improve quality of life, activities of daily living, care burden and cognitive functioning in institutionalized older adults with dementia – a randomized controlled trial study protocol

**DOI:** 10.1186/s12877-018-0874-4

**Published:** 2018-08-14

**Authors:** Marelle Heesterbeek, Eddy A. Van der Zee, Marieke J. G. van Heuvelen

**Affiliations:** 1 Molecular Neurobiology, Groningen Institute for Evolutionary Life Sciences (GELIFES), University of Groningen, Nijenborgh 7, 9747 AG Groningen, The Netherlands; 2 Center for Human Movement Sciences, University of Groningen, University Medical Center Groningen, Groningen, The Netherlands

**Keywords:** Dementia, Motion simulation, Whole body vibration, Randomized controlled trial, Quality of life, Activities of daily living, Care burden, Cognition, Physical function

## Abstract

**Background:**

Dementia affects cognitive functioning, physical functioning, activities of daily living (ADLs), and quality of life (QOL). Pharmacological treatments to manage, cure or prevent dementia remain controversial. Therefore development of non-pharmacological approaches to prevent, or at least delay the onset and progression of dementia is urgently needed. Passive exercise is proposed to be such a non-pharmacological alternative. This study primarily aims to investigate the effects of three different forms of passive exercise on QOL and ADLs of institutionalized patients with dementia. The secondary aims are to assess the effects of three different forms of passive exercise on cognitive functioning and physical functioning of institutionalized patients with dementia as well as on care burden of both the primary formal and primary informal caregivers of these patients.

**Methods:**

This is a multicenter randomized controlled trial. Three forms of passive exercise are distinguished; motion simulation (MSim), whole body vibration (WBV), and a combination of both MSim + WBV. Intervention effects are compared to a control group receiving regular care. Institutionalized patients with dementia follow a six-week intervention program consisting of four 4–12 min sessions a week.

The primary outcome measures QOL and ADLs and secondary outcome measure care burden are assessed with questionnaires filled in by the primary formal and informal caregivers of the patient. The other secondary outcome measures cognitive and physical functioning are assessed by individual testing. The four groups are compared at baseline, after 6 weeks of intervention, and 2 weeks after the intervention has ended.

**Discussion:**

This study will provide insight in the effects of different forms of passive exercise on QOL, ADLs, cognitive and physical functioning and care burden of institutionalized patients with dementia and their primary formal and informal caregivers. The results of this study might support the idea that passive exercise can be an efficient alternative for physical activity for patients not able to be or stay involved in active physical exercise.

**Trial registration:**

The Netherlands National Trial Register (NTR6290). Retrospectively registered 29 March 2017.

## Background

Dementia is a life changing condition that is characterized by progressive cognitive decline and motor deficits, often leading to psychological symptoms, decline in quality of life (QOL) and the ability to perform activities of daily living (ADLs). These disabilities lead to loss of autonomy and need for (in)formal care, in most cases even institutionalization is required. Institutionalization, however, often results in a further decline in QOL of the patient [1].

To date no cure for dementia is developed. Pharmacological treatments have been unsuccessful and often have many side effects. Therefore, over the past few years there has been a growing interest in non-pharmacological interventions to limit the adverse effects of dementia. Physical exercise is one example of such a non-pharmacological intervention. In multiple studies positive effects of physical exercise on QOL, ADLs, physical and cognitive functioning of patients with dementia were found [2–7]. Physical exercise thus seems to be an effective treatment strategy to limit the adverse effects of dementia. However, due to physical decline, behavioral problems and limited time of caregivers to accompany patients, engaging in physical exercise is not possible for most institutionalized dementia patients.

**Fig. 1:**
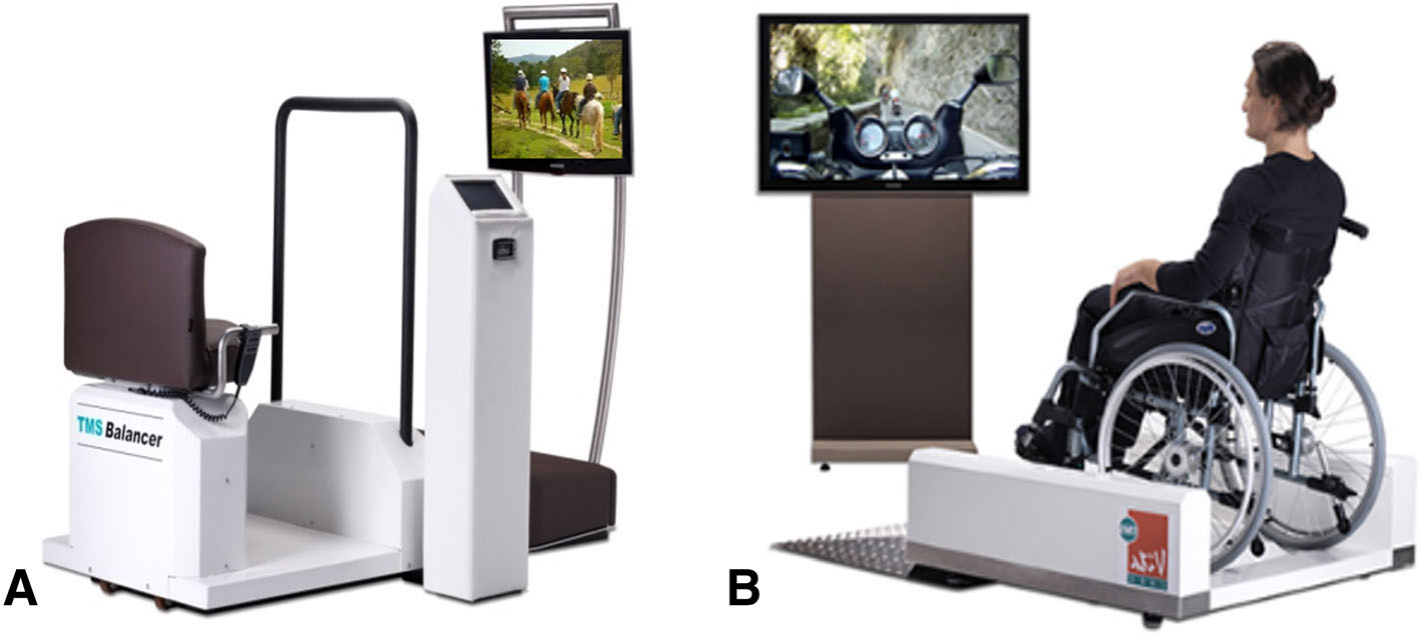
Motion simulation devices **a** The balancer with a chair, screen and control panel, and **b** the wheelchair pod with a wheelchair platform and television screen (identical control panel as in Fig. 1a is not depicted). Both platforms are used to provide the MSim, WBV and MSim + WBV intervention sessions

It is proposed that passive exercise can be an efficient alternative for physical exercise to enhance QOL and ADLs in institutionalized patients with dementia. Three forms of passive exercise that can be employed with a robotized movement platform (Fig. 1a, b) are distinguished; motion simulation (MSim), whole body vibration (WBV), and a combination of both MSim + WBV.

During MSim movies of multiple activities from various perspectives are shown on a television screen. During these movies the robotized platform moves synchronically with the activities on the screen. Also matching sounds and music are played. Altogether MSim stimulates the visual, auditory, tactile, and proprioceptive system of a participant. To the best of our knowledge, to date MSim in this specific form has not yet been studied. However, other studies that employed either visual or auditory stimulation showed that video and music interventions can improve alertness and happiness, promote social behavior, and reduce behavioral disturbances as well agitated behavior of patients with dementia [8–10].

During WBV, participants are exposed to mild vibrations via contact with a vibration source. Thereby, WBV provides tactile and proprioceptive input to the participant. The intensity of WBV can be controlled by adjusting the frequency, the amplitude (peak to peak displacement), and the time of exposure. Multiple studies reported that mild vibrations (30–40 Hz) can improve physical performance and health related components such as increased muscle strength, mobility, balance and lower blood pressure [11–14]. Furthermore WBV was also found to improve cognitive functioning, for example attention and inhibition of schoolchildren, young adults and persons with attention deficit hyperactive disorder [15, 16].

The exact working mechanisms of WBV and MSim remain unknown. However, we assume that the following mechanisms may play a role. WBV can induce brain activation, especially in the sensory (motor) cortex. Skin mechanoreceptors, e.g. Meissner corpuscles, are sensitive for 30 Hz stimulation and activation of these receptors leads to activation of the spinothalamic pathway and the medial lemniscal pathway, both ending in the sensory motor cortex [17–19]. The visual, auditory and sensory stimuli of MSim on the other hand can elicit brain activation in the different layers of the visual cortex, the cochlear nucleus in the brainstem and to a lesser extent than WBV in the sensory motor cortex [18]. Moreover, it is thought that the large translational and rotational movements of MSim will also increase signaling of the vestibular system to for example the cerebellum, thalamus and reticular formation in order to maintain an upright posture [20]. Activation of the above mentioned areas and pathways may induce increased blood flow in and synaptic strengthening of these specific areas and pathways. Furthermore neurite outgrowth and functioning of underlying neurotransmitter systems can be enhanced [21–25]. For both MSim and WBV association areas will receive input from the activated sensory areas, causing a diffuse activation of brain regions [26]. We believe that during MSim a more diverse sensory integration of the different stimuli will take place, while during WBV primarily the sensory (motor) cortex processing vibration stimuli will be activated. When combining MSim and WBV it is thought that the combination of high activation in the sensory (motor) cortex (WBV) and sensory integration of the visual, auditory and sensory stimuli (MSim) will enhance the effects seen for WBV and MSim alone.

Although MSim is already being used in multiple health care settings, any effects of MSim alone or combined with WBV have not yet been established. The current study primarily aims to investigate the effects of the three different forms of passive exercise on QOL and ADLs of institutionalized patients with dementia. The secondary aims are to assess the effects of the three different forms of passive exercise on cognitive functioning and physical functioning of institutionalized patients with dementia as well as on care burden of both the primary formal and primary informal caregivers of these patients. Cost-effectiveness of the interventions will also be analyzed. It is hypothesized that MSim and WBV, due to their own specific working mechanisms, both will lead to an improvement in QOL, ADLs and cognitive functioning, but that the combination of MSim and WBV will lead to a stronger cognitive effect as a results of complementary working mechanisms. In addition, it is hypothesized that improvement in ADLs and improved cognition will lead to more independent and better daily functioning of the patient, causing a reduction in need for care and consequently lower the care burden of the primary formal and informal caregiver..

## Methods

### Study design and setting

This is a single blind randomized controlled trial. The study will be conducted in the closed wards of nursing homes in the north of the Netherlands. The effects of three different types of passive exercise will be studied and compared to a control group receiving regular care.

The protocol follows the Standard Protocol Items: Recommendations for Interventions Trials (SPIRIT) 2013 statement [27]. The study protocol is approved by the medical ethics committee of the University Medical Center Groningen (the Netherlands), according to the principles of the Declaration of Helsinki, and is registered with The Netherlands National Trial Register (NTR6290, http://www.trialregister.nl).

### Participants and eligibility criteria

Institutionalized older adults with dementia will be included in this multicenter, single blind randomized controlled trial. People are eligible if they are officially diagnosed with some form of dementia, aged 65 years or older, not physically active for more than 10 min a day. Participants will be excluded if they have a contra-indication for exercise, have a serious auditory disorder, are color blind, and/or excessively use alcohol or drugs.

### Study procedures

The participants of this study will be recruited via the medical staff of nursing homes. The medical staff and nurses, who are informed about the aim and procedure of the study, select potential participants within the different wards of the nursing homes. The potential participants and their legal representatives then receive an information letter with informed consent. The legal representatives of the potential participants can provide written informed consent, and in addition patients will have to orally agree to participate. After informed consent is given by the legal representatives, participants will be screened for eligibility.

**Fig. 2:**
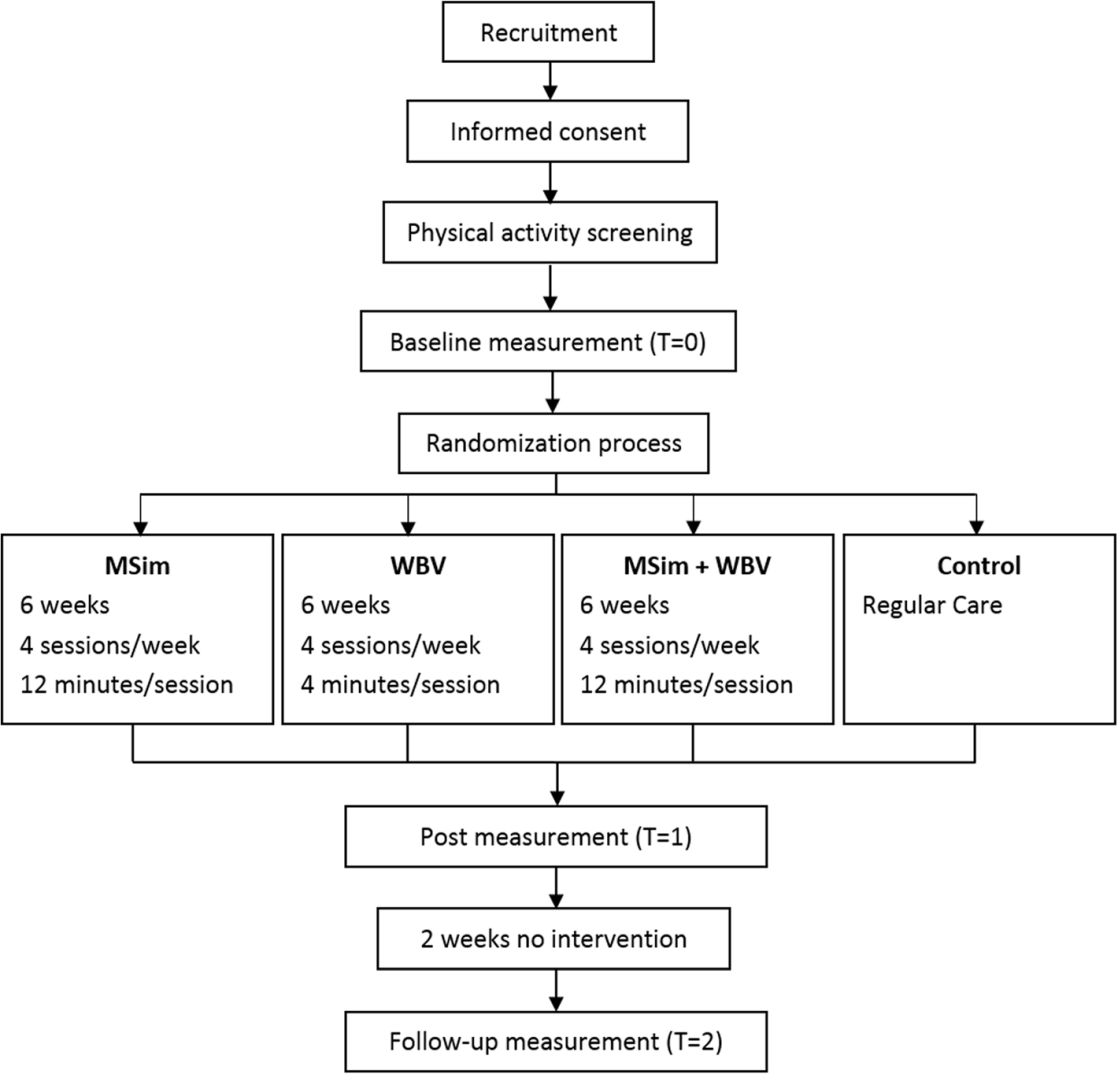
Flowchart of the study processes

Measurements for cognitive and physical function are administrated by trained research assistants who are blinded for group allocation. Questionnaires for QOL, ADLs and care burden are given or send to respectively the primary formal and primary informal caregiver. Tests and questionnaires for all outcome measures are assessed at baseline (T0), after the intervention period of six weeks (T1) and two weeks after the intervention has ended (T2). All test moments of a given participant are performed at the same time of the day and are assessed by the same research assistant. Also the questionnaires for both the primary formal and primary informal caregiver are filled in by the same person on each given time point. An overview of the enrollment and study design is given in Fig. 2.

#### Randomization

After baseline measurements and stratification for gender, MMSE score, age and nursing home, participants are randomly assigned (1:1:1:1 allocation ratio) to one of the four groups (MSim, WBV, MSim + WBV or regular care which serves as the control condition) by using random numbers. The randomization procedure is performed by a blinded scientist who is not related to the study.

#### Interventions

All participants in the intervention groups receive the intervention four times a week for six consecutive weeks. During these six weeks, participants in the control group receive regular care.

The first intervention group receives MSim. During a session, three short, real life movies of approximately four minutes are shown of multiple activities (e.g. motor riding, dancing or horse riding) and from various perspectives. Matching music and sounds are played and the platform moves synchronically with the movies. This way, the participant on the platform moves in a passive way and is stimulated multisensory by means of visual, auditory, tactile and proprioceptive stimuli.

During WBV, the platform vibrates with a 30 Hz frequency and an amplitude of 1–2 mm. In an earlier study it was shown that these vibrations can safely be applied in old adults [28]. The duration of the WBV sessions is set to four minutes. During the WBV session a stationary motorcycle with idling engine is shown on the screen and matching sounds are played.

For the MSim + WBV intervention, the former two forms of passive exercise are combined. During 12 min participants alternately receive MSim (4 min) and WBV (2 min).

All forms of passive exercise will be applied using two commercially available motion simulation devices (the balancer, Fig. 1a and the wheelchair pod, Fig. 1b). During the sessions, participants are asked to take place on either one of the platforms and focus on the television screen. Hands are placed on the sidebars of the balancer or the wheelchair. Preferably the participant is seated as upright as possible.

The types of movies,the movement intensity of the platform and vibration intensity are documented. After each session the participant will be asked how much he/she enjoyed the session. Scores can be given on a scale ranging from zero to ten, with zero meaning they did not enjoy the session at all and ten meaning that they really enjoyed the session. Also, in the MSim and MSim + WBV group, the participants are asked which movies of the session they liked the most. For the MSim and MSim + WBV group respectively a top 3 or top 2 is documented. In the first week, at least one movie of each activity will be shown to the participant. After the first week, if any preferences are known for a participant, movies shown in the remaining sessions are chosen based on these preferences.

### Outcome measures

#### QOL and ADLs

To assess QOL and ADLs a series of questionnaires are used.

The EQ-5D-5 L [29] is filled in by the primary formal caregiver. It consists of five 5-point Likert scale questions, and is used to measure health-related quality of life based on five different domains (mobility, self-care, usual activities, pain/discomfort, anxiety/depression). An index value can be computed using the Crosswalk Index Value Calculator [30] with the value set from the Netherlands; an index value closer to 1 resembles a better quality of life. This index value of the EQ-5D-5 L can also be used for cost effectiveness analyses.

The Qualidem [31] is also filled in by the primary formal caregiver. The questionnaire consists of 40 items on a 4-point Likert scale and is specifically developed to quantify QOL in institutionalized patients with dementia. The following subscales can be distinguished: care relationship, positive affect, negative affect, restless tense behavior, positive self-image, social relations, social isolation, feeling at home, having something to do and other. A sum score between 0 and 120 can be obtained, a higher score reflects a better quality of life.

The Older Persons and Informal Caregivers Survey Minimum DataSet (TOPICS-MDS) is a set of brief, standardized questionnaires filled in by the primary informal caregiver, if possible in consultation with the participant [32, 33]. Next to demographic characteristics, outcome measures are comorbidity, quality of life, mood, functional limitations, mental health, social functioning, care use and care burden. For QOL the TOPIC-MDS includes the EQ-3D-3 L and two questions adapted from the RAND-36 [34, 35]. For ADLs the TOPICS-MDS includes a modified version of the Katz Index and an additional indicator of mobility [36]. Respondents are asked if the participant requires assistance for six basic functions (e.g. dressing, eating and bathing), and seven instrumental functions (e.g. grooming and taking medication). Responses are rated on a binary scoring system (dependent = 1; independent = 0) and summated, with higher scores representing greater functional limitations [32].

An adapted version of the Barthel-Index [37] is filled in by the primary formal caregiver and is used to measure ADL functioning and mobility. The adapted version is an observation scale that consists of ten items on a 5-points Likert scale. Sum scores can range between 0 and 90, with higher scores representing greater ADL functioning and mobility.

#### Cognitive functioning

To evaluate the effects of passive exercise on cognitive functioning a neuropsychological test battery that covers de subdomains global cognitive functioning, executive functioning and verbal memory is used.

Global cognitive functioning is measured using the Mini Mental State Examination (MMSE) [38].The MMSE is a brief 20 item questionnaire test that is used to screen for cognitive impairment. The items refer to orientation, attention, memory, recall, and processing verbal and written information. Scores on this test can range from 0 to 30, a higher score reflects a higher level of global cognitive function.

To measure attention the Deary-Liewald simple reaction time task (SRT) [39] is used. During the SRT participants have to press a key, as quickly as possible in response to a single stimulus. The stimulus consists of a black cross appearing in a white square. Five practice trials are given (the practice trial can be repeated up to a maximum of 3 times to make sure the participant fully understands the task), after which instructions are repeated. The official test consists of a minimum of 15 trials. The test stops if the participant has 15 correct responses, correct being a response within the time limits of 150–3000 ms. Means and standard deviations are measured for each participant. Also the number of correct and incorrect (premature and anticipated) responses is measured.

The Stroop test is used to measure attention and inhibition [40]. It consists of three subtasks, all performed as quickly and accurately as possible within 45 s. For the first subtask a card is used with names of 4 colors (‘blue’, ‘green’, ‘red’, ‘yellow’) printed on it in black ink. Participants have to name the right words in the right sequence (left-right, top-bottom), as fast as possible. For the second subtask a card with colored blocks (blue, green, red, and yellow) is used. Participants have to name the right color of the block in the right sequence, as fast as possible. During the third subtask a card with names of colors (‘blue’, ‘green’, ‘red’, ‘yellow’) printed in opposite colors (e.g. ‘red’ written in blue ink or ‘yellow’ written in green ink) is presented to the participant. The participant now has to name the ink of as many words as possible in the right sequence. The score for each subtask consists of the number and the accuracy (%) of correctly named words or colors, a higher number and percentage reflects better inhibition.

The digit span forward (DSf) and backward (DSb) tests are used to measure respectively verbal short term memory and working memory [41]. During the DSf series of verbally presented digits are asked to be repeated. While during the DSb series of verbally presented digits are asked to be repeated in reverse order. In both tests the number of digits increases by one digit every two trials. The test is stopped when the participants fails to correctly repeat two consecutive series. The score is the number of successful repeated series, with higher scores indicating a better performance.

The short version of the Trail Making Test part A (TMT A) is used to measure visuomotor speed and attention. Participants have to draw a line between encircled numbers in increasing order (1–14) [42]. The time to complete the task is recorded. Lower scores indicate a better performance.

To assess divergent thinking and language skills, both the phonemic fluency and semantic fluency tests are used. For the phonemic fluency test participants are asked to name words starting with a specific letter (‘D’, ‘A’ and ‘T’) [43]. For each letter they have one minute to name as many words as possible. It is not allowed to use (part of) a word multiple times (e.g. snow, snowmen, snowball), names or digits. The total number of correct words from all three trials counts as score. A higher score reflects better performance. For the semantic fluency participants are asked to name as many ‘professions’ in one minute. The outcome measure is the total number of professions, with a higher score indicating a better performance.

#### Physical functions

For participants who are not wheelchair bound the following tests are used to assess physical functioning in multiple domains.

The Timed Up & Go test (TUG) is used to measure functional mobility [44]. The participant is instructed to rise from a chair, walk 3 m, make a turn, walk back and sit down in the chair, as fast as possible, but without running. Participants are allowed to use their hands while standing up and also walking devices are allowed during test performance. Two trials are performed and the average time is the outcome, lower scores implying better functional mobility.

The FICSIT-4 measures static balance [45]. Participants are asked to perform a stance with two feet parallel, semi tandem, tandem and on a single leg. The participant has to hold every stance for 10 s. If a stance cannot be held for 10 s, the test stops. Scores can range from 0 to 5, with higher scores indicating better performance.

A 6-m walking test is used to assess walking speed. Participants are instructed to walk 10 m as fast as possible, but without running. Time registration starts after 2 m and stops 2 m before the end of the 10 m track. The use of a walking aid is allowed. For each participant three attempts are timed, the mean time of three trials is used as score, a lower score implies a better performance.

#### Care burden

Care burden of both the primary informal caregiver as well as the primary formal caregiver is assessed with a set of brief questionnaires.

The Caregiver Strain Index (CSI) is filled in by the primary informal caregiver and evaluates (over)load of the family caregiver on 13 yes/no items [46].

The short version of the Zarit Burden Interview (ZBI) evaluates care burden of the primary informal caregiver [47]. It consists of 12 5-point Likert scale questions and is widely used in dementia caregiving research. Scores can range between 0 and 48. Higher scores represent higher care burden.

Next to the short standardized questionnaires, care burden of both the primary formal caregiver as well as the primary informal caregiver is evaluated with an adapted Borg scale (originally used in sports as a measure of perceived exertion). Scores can range between zero and fourteen, zero indicating no care burden at all and fourteen indicating maximum care burden.

#### Cost-effectiveness

To evaluate the cost-effectiveness of the passive exercise interventions, QOL outcomes and costs need to be determined. The outcomes of the EQ-5D-5 L will be used to calculate quality adjusted life years (QALYs). QALYs are expressed as a number between 0 and 1, where 0 indicates death and 1 indicates optimal QOL, multiplied with the life expectancy of the person. To estimate the costs of the intervention, the hours of intervention and the costs of the MSim platform will be included. Cost-effectiveness will be specified as the incremental cost-effectiveness ratio (ICER), which is defined by the cost per incremental QALY. Separate cost-effectiveness ratios will be calculated for each type of passive exercise, compared to regular care using the following formulae: ICER = (Costs Intervention – Costs Control)/(Effect Intervention – Effect Control).

### Sample size calculation

Sample size is calculated with Sample Power 3. Since there is no data available on MSim or any comparable intervention, the sample size is calculated with an effect size estimation based on results of pilot studies on the chronic effects of WBV on cognition. Two pilot studies used the Stroop test score to evaluate these effects and resulted in an effect size of f = 0.22 [unpublished observations]. Power analyses with this value and use of repeated measures ANOVA, alpha 5%, power 80% and expected drop-out of 15% results in a minimal sample size of 49 participants per group.

Some additional remarks are needed to explain this sample size calculation. Since pilot data for QOL and ADLs are not available, the sample size estimation is based on the secondary outcome measure cognition and not on the primary outcome measures quality of life and daily functioning. Moreover, we are fully aware of the fact that with the calculated sample size additional subgroup analyses lack power. Including these analyses in the sample size calculation would result in unrealistic high sample sizes which would be practically impossible to realize due to factors like available budget, duration of the study and available number of potential participants. Additional analyses will be performed but will be considered exploratory.

### Statistics

For each group, descriptive statistics of the sample’s sociodemographic and clinical characteristics at baseline will be determined. Analyses of covariance (continuous, normally distributed data) or non-parametric alternatives (ordinal, non-normally distributed data) will be used to examine differences between groups at baseline.

Since all primary outcome measures and some of the secondary outcome measures result from questionnaires, they will be of ordinal level of measurement and probably have a skewed distribution. Therefore, non-parametric tests will be used to analyze the data from the questionnaires. Gain scores (post-test minus pretest and follow-up test minus pretest/posttest) will be calculated and the differences between the groups will be analyzed using Kruskall-Wallis tests followed by Bonferoni corrected Mann-Whitney tests for paired comparisons.

The outcome measures related to cognitive and physical function will be of continuous level. If the data satisfy the assumptions, analyses of covariance will be used with scores on cognitive tests at T1/T2 as dependent variables, pretest scores as covariates and group (experimental groups, control group) as between-subjects factor. If the data do not meet the assumptions repeated measures analyses of variance or non-parametric methods with gain scores will be considered. Post-hoc tests will be performed with Bonferoni corrections for multiple comparisons. The data will be analyzed both according to intention-to-treat method (irrespective of adherence to intervention) as per-protocol (for selection of participants with sufficient adherence).

Missing data of items within questionnaires are substituted as prescribed for the individual questionnaires. If no prescription is available, the maximum likelihood method is used to substitute the missing values. Based on the internal consistency of the questionnaires, the maximum number of substituted values will be determined (Cronbach’s alpha >.80, max 50% of the items can be substituted).

A *p*-value of < 0.05 will be used to assess statistical significance. For all tests power and effect size calculations will be performed. Effect sizes will be calculated with Cohen’s d to measure the magnitude of difference of gain score measurements between each experimental and control group. Values benchmarking small, medium and large effect sizes: d = .20, d = .50, d = .80 [48].

## Discussion

MSim and WBV are new and promising interventions for patients not able to be or stay involved in physical exercise. MSim and WBV are currently implemented in a variety of health care settings. However, there is limited evidence, if at all, of the clinical and cost-effectiveness of these interventions. To the best of the authors knowledge, the current trial is the first to study the proposed three forms of passive exercise in institutionalized patients with dementia.

The targeted population is a major strength of the study. The current in- and exclusion criteria ensure that the interventions are applied to a wide variety of dementia patients, thereby enhancing the generalizability of the results. Furthermore, the variety of movies and the possibility to adjust the intensity of the movements and sounds used for MSim makes that MSim can be personalized to a great extent. Consequently high adherence rates are expected in the present study.

MSim is a complex multi component intervention. Outcomes are expected in a wide range of variables. Therefore, in addition to the primary outcome measures, several secondary outcome measures are included, which have the potential to capture possible effects. This is a great advantage of the study. However we are also aware that the multiple comparisons leading from this variety of outcome measures will lead to a lack of statistical power of these comparisons. Therefore such additional analyses will be considered exploratory.

One of the possible threats and limitations of the current study is the use of questionnaires for assessment of the primary outcome measures. To determine QOL and ADLs of the patients, questionnaires that are filled in by the primary formal caregiver and the primary informal caregiver are used. It is impossible to blind these caregivers during the intervention. The subjective nature of these measures might influence the outcomes. However, the caregivers are not informed about the hypotheses of the study. Therefore, no bias within the intervention groups is to be expected. Another possible limitation of this study is that due to the multi component nature of MSim and MSim + WBV, no indisputable conclusions can be drawn about which specific component(s) caused the effects that might be found after the intervention period. It will be possible to compare the different interventions with each other. However no decisive answers can be given to the question which sensory stimuli caused the potentially found effects. Moreover, since the trial lacks a social control group, any assumptions of the social component of the intervention causing potential effects cannot be falsified. However, effect sizes of the intervention groups of the current trial can be compared to effect sizes found in exercise studies in the same population in which social controls are included.

## Abbreviations

ADLs: Activities of daily living
CSI: Caregiver strain index
DSb: Digit span backward
DSf: Digit span forward
MMSE: Mini mental state examination
MSim: Motion simulation
QALYs: Quality adjusted life years
QOL: Quality of life
SRT: Simple reaction time
TMT A: Trail making test part A
TOPICS-MDS: The older persons and informal caregivers survey minimum dataset
TUG: Timed up & go test
WBV: Whole body vibration
ZBI: Zarit burden interview

## References

[1] Cobo CMS (2014). The influence of institutionalization on the perception of autonomy and quality of life in old people. Rev Esc Enferm USP.

[2] Bossers WJ, Van der Woude LHV, Boersma F, Hortobágyi T, Scherder EJ, van Heuvelen MJ (2015). A 9-week aerobic and strength training program improves cognitive and motor function in patients with dementia: a randomized, controlled trial. Am J Geriatr Psychiatry.

[3] Dechamps A, Diolez P, Thiaudière E, Tulon A, Onifade C, Vuong T, Helmer C, Bourdel-Marchasson I (2010). Effects of exercise programs to prevent decline in health-related quality of life in highly deconditioned institutionalized elderly persons: a randomized controlled trial. Arch Intern Med.

[4] Toots A, Littbrand H, Lindelöf N, Wiklund R, Holmberg H, Nordström P, Lundin-Olsson L, Gustafson Y, Rosendahl E (2016). Effects of a high-intensity functional exercise program on dependence in activities of daily living and balance in older adults with dementia. J Am Geriatr Soc.

[5] Venturelli M, Scarsini R, Schena F (2011). Six-month walking program changes cognitive and ADL performance in patients with Alzheimer. Am J Alzheimers Dis Other Demen.

[6] Liu-Ambrose T, Nagamatsu LS, Graf P, Beattie BL, Ashe MC, Handy TC (2010). Resistance training and executive functions: a 12-month randomized controlled trial. Arch Intern Med.

[7] Potter R, Ellard D, Rees K, Thorogood M (2011). A systematic review of the effects of physical activity on physical functioning, quality of life and depression in older people with dementia. Int J Geriatr Psychiatry.

[8] Cohen-Mansfield J, Marx MS, Dakheel-Ali M, Thein K (2015). The use and utility of specific nonpharmacological interventions for behavioral symptoms in dementia: an exploratory study. Am J Geriatr Psychiatr.

[9] Casby J, Holm M (1994). The effect of music on repetitive disruptive vocalizations of persons with dementia. Am J Occup Ther.

[10] Lord TR, Garner JE (1993). Effects of music on Alzheimer patients. Percept Mot Skills.

[11] Ebid AA, Ahmed MT, Eid MM, Mohamed MSE (2012). Effect of whole body vibration on leg muscle strength after healed burns: a randomized controlled trial. Burns.

[12] Figueroa A, Gil R, Wong A, Hooshmand S, Park SY, Vicil F, Sanchez-Gonzalez MA (2012). Whole-body vibration training reduces arterial stiffness, blood pressure and sympathovagal balance in young overweight/obese women. Hypertens Res.

[13] Zhang L, Weng C, Liu M, Wang Q, Liu L, He Y (2014). Effect of whole-body vibration exercise on mobility, balance ability and general health status in frail elderly patients: a pilot randomized controlled trial. Clin Rehabil.

[14] Turbanski S, Haas CT, Schmidtbleicher D, Friedrich A, Duisberg P (2005). Effects of random whole-body vibration on postural control in Parkinson's disease. Res Sports Med.

[15] Fuermaier ABM, Tucha L, Koerts J, van Heuvelen MJG, van der Zee EA, Lange KW, Tucha O (2014). Good vibrations - effects of whole body vibration on attention in healthy individuals and individuals with ADHD. PLoS One.

[16] Regterschot GRH, Van Heuvelen MJ, Zeinstra EB, Fuermaier AB, Tucha L, Koerts J, Tucha O, Van Der Zee EA (2014). Whole body vibration improves cognition in healthy young adults. PLoS One.

[17] Dykes RW (1983). Parallel processing of somatosensory information: a theory. Brain Res Rev.

[18] Martin J. Neuroanatomy text and atlas. New York: McGraw Hill professional; 2012.

[19] Talbot WH, Darian-Smith I, Kornhuber HH, Mountcastle VB (1968). The sense of flutter-vibration: comparison of the human capacity with response patterns of mechanoreceptive afferents from the monkey hand. J Neurophysiol.

[20] Angelaki DE, Cullen KE (2008). Vestibular system: the many facets of a multimodal sense. Annu Rev Neurosci.

[21] Koike Y, Tutida R, Hayashi Y, Yamanishi Y, Kano Y. Low-Frequency, Whole Body Vibration Induced Neurite Outgrowth by Pc12m3 Cells with Impaired Nerve Growth Factor-Induced Neurite Outgrowth. J Nov Physiother. 2015;5(1):249.

[22] Acquas E, Wilson C, Fibiger HC (1996). Conditioned and unconditioned stimuli increase frontal cortical and hippocampal acetylcholine release: effects of novelty, habituation, and fear. J Neurosci.

[23] Doig NM, Magill PJ, Apicella P, Bolam JP, Sharott A (2014). Cortical and thalamic excitation mediate the multiphasic responses of striatal cholinergic interneurons to motivationally salient stimuli. J Neurosci.

[24] Van der Zee E, Riedel G, Rutgers E, De Vries C, Postema F, Venema B (2010). Enhanced neuronal activity in selective brain regions of mice induced by whole body stimulation. Federation Eur Neurosci Societies Abstract.

[25] Heesterbeek M, Jentsch M, Roemers P, Keijser JN, Toth K, Nyakas C, Schoemaker RG, van Heuvelen MJG, van der Zee EA (2017). Whole body vibration enhances choline acetyltransferase-immunoreactivity in cortex and amygdale. J Neurology Translational Neurosci.

[26] Braak H, Braak E, Yilmazer D, Bohl J (1996). Topical review: functional anatomy of human hippocampal formation and related structures. J Child Neurol.

[27] Chan A, Tetzlaff JM, Altman DG, Laupacis A, Gøtzsche PC, Krleža-Jerić K, Hróbjartsson A, Mann H, Dickersin K, Berlin JA (2013). SPIRIT 2013 statement: defining standard protocol items for clinical trials. Ann Intern Med.

[28] Bruyere O, Wuidart M, Di Palma E, Gourlay M, Ethgen O, Richy F, Reginster J (2005). Controlled whole body vibration to decrease fall risk and improve health-related quality of life of nursing home residents. Arch Phys Med Rehabil.

[29] Herdman M, Gudex C, Lloyd A, Janssen M, Kind P, Parkin D, Bonsel G, Badia X (2011). Development and preliminary testing of the new five-level version of EQ-5D (EQ-5D-5L). Qual Life Res.

[30] Van Hout B, Janssen M, Feng Y, Kohlmann T, Busschbach J, Golicki D, Lloyd A, Scalone L, Kind P, Pickard AS (2012). Interim scoring for the EQ-5D-5L: mapping the EQ-5D-5L to EQ-5D-3L value sets. Value Health.

[31] Ettema TP, Dröes R, de Lange J, Mellenbergh GJ, Ribbe MW (2007). QUALIDEM: development and evaluation of a dementia specific quality of life instrument. Scalability, reliability and internal structure. Int J Geriatr Psychiatry.

[32] Lutomski JE, Baars MA, Schalk BW, Boter H, Buurman BM, den Elzen WP, Jansen AP, Kempen GI, Steunenberg B, Steyerberg EW (2013). The development of the older persons and informal caregivers survey minimum DataSet (TOPICS-MDS): a large-scale data sharing initiative. PLoS One.

[33] van den Brink D, Lutomski JE, Qin L, den Elzen WP, Kempen GI, Krabbe PF, Steyerberg EW, Muntinga M, Moll van Charante EP, Bleijenberg N (2015). TOPICS-MDS: Veelzijdige bron voor wetenschappelijke en maatschappelijke kennisgeneratie ten behoeve van de ouderenzorg. Tijdschr Gerontol Geriatr.

[34] Van der Zee K, Sanderman R: RAND-36. Groningen: Northern Centre for Health Care Research, University of Groningen, the Netherlands 1993, 28.

[35] Hays RD, Morales LS (2001). The RAND-36 measure of health-related quality of life. Ann Med.

[36] Weinberger M, Samsa GP, Schmader K, Greenberg SM, Carr DB, Wildman DS (1992). Comparing proxy and patients' perceptions of patients' functional status: results from an outpatient geriatric clinic. J Am Geriatr Soc.

[37] Shah S, Vanclay F, Cooper B (1989). Improving the sensitivity of the Barthel index for stroke rehabilitation. J Clin Epidemiol.

[38] Folstein MF, Folstein SE, McHugh PR (1975). “mini-mental state”: a practical method for grading the cognitive state of patients for the clinician. J Psychiatr Res.

[39] Deary IJ, Liewald D, Nissan J (2011). A free, easy-to-use, computer-based simple and four-choice reaction time programme: the Deary-Liewald reaction time task. Behav Res Methods.

[40] Stroop JR (1935). Studies of interference in serial verbal reactions. J Exp Psychol.

[41] Wechsler D. WAIS-III: Wechsler adult intelligence scale. New York: psychological corporation; 1997.

[42] Reitan RM (1971). Trail making test results for normal and brain-damaged children. Percept Mot Skills.

[43] Schmand B, Groenink S, Van den Dungen M (2008). Letterfluency: psychometrische eigenschappen en Nederlandse normen. Tijdschr Gerontol Geriatr.

[44] Podsiadlo D, Richardson S (1991). The timed “up & go”: a test of basic functional mobility for frail elderly persons. J Am Geriatr Soc.

[45] Rossiter-Fornoff JE, Wolf SL, Wolfson LI, Buchner DM (1995). A cross-sectional validation study of the FICSIT common data base static balance measures. Frailty and injuries: cooperative studies of intervention techniques. J Gerontol A Biol Sci Med Sci.

[46] van Exel NJ, Op Reimer WJMS, Brouwer WB, van den Berg B, Koopmanschap MA, van den Bos GAM (2004). Instruments for assessing the burden of informal caregiving for stroke patients in clinical practice: a comparison of CSI, CRA, SCQ and self-rated burden. Clin Rehabil.

[47] Bédard M, Molloy DW, Squire L, Dubois S, Lever JA, O'Donnell M (2001). The Zarit burden interview a new short version and screening version. Gerontologist.

[48] Cohen J (1988). The effect size index: d. Statistical power analysis for the behavioral sciences.

